# Differential Effects of Purinergic Signaling in Gastric Cancer-Derived Cells Through P2Y and P2X Receptors

**DOI:** 10.3389/fphar.2019.00612

**Published:** 2019-06-13

**Authors:** María José Hevia, Patricio Castro, Katherine Pinto, Mauricio Reyna-Jeldes, Felipe Rodríguez-Tirado, Claudia Robles-Planells, Sebastián Ramírez-Rivera, Juan Andrés Madariaga, Felipe Gutierrez, Javier López, Marcelo Barra, Erwin De la Fuente-Ortega, Giuliano Bernal, Claudio Coddou

**Affiliations:** ^1^Departamento de Ciencias Biomédicas, Facultad de Medicina, Universidad Católica del Norte, Coquimbo, Chile; ^2^Departamento de Fisiología, Facultad de Ciencias Biológicas, Universidad de Concepción, Concepción, Chile; ^3^Departamento de Biología, Facultad de Ciencias, Universidad de Chile, Santiago, Chile; ^4^Hospital San Pablo, Coquimbo, Chile

**Keywords:** gastric cancer, P2Y receptor, P2X receptor, ATP, uridine triphosphate (UTP), paracrine action

## Abstract

Gastric cancer (GC) is the one of the most prevalent cancers and one of the leading causes of cancer-induced deaths. Previously, we found that the expression of purinergic P2Y_2_ receptor (P2Y_2_R) is increased in GC samples as compared to adjacent healthy mucosa taken from GC-diagnosed patients. In this work, we studied in detail purinergic signaling in the gastric adenocarcinoma-derived cell lines: AGS, MKN-45, and MKN-74, and compared them to a nontumoral epithelial cell line: GES-1. In GC-derived cells, we detected the expression of several purinergic receptors, and found important differences as compared to GES-1 cells. Functional studies revealed a strong contribution of P2Y_2_Rs in intracellular calcium increases, elicited by adenosine-triphosphate (ATP), uridine-triphosphate (UTP), and the P2Y_2_R agonist MRS2768. Responses were preserved in the absence of extracellular calcium and inhibited by P2Y_2_R antagonists. In GES-1 cells, ATP and UTP induced similar responses and the combination of P2X and P2Y receptor antagonists was able to block them. Proliferation studies showed that ATP regulates AGS and MKN-74 cells in a biphasic manner, increasing cell proliferation at 10–100 μM, but inhibiting at 300 μM ATP. On the other hand, 1–300 μM UTP, a P2Y_2_R agonist, increased concentration-dependent cell proliferation. The effects of UTP and ATP were prevented by both wide-range and specific purinergic antagonists. In contrast, in GES-1 cells ATP only decreased cell proliferation in a concentration-dependent manner, and UTP had no effect. Notably, the isolated application of purinergic antagonists was sufficient to change the basal proliferation of AGS cells, indicating that nucleotides released by the cells can act as paracrine/autocrine signals. Finally, in tumor-derived biopsies, we found an increase of P2Y_2_R and a decrease in P2X4R expression; however, we found high variability between seven different biopsies and their respective adjacent healthy gastric mucosa. Even so, we found a correlation between the expression levels of P2Y_2_R and P2X4R and survival rates of GC patients. Taken together, these results demonstrate the involvement of different purinergic receptors and signaling in GC, and the pattern of expression changes in tumoral cells, and this change likely directs ATP and nucleotide signaling from antiproliferative effects in healthy tissues to proliferative effects in cancer.

## Introduction

Cancer, or malignant tumors, comprises a group of diseases that involve abnormal cell growth and proliferation and is a leading cause of death worldwide. Several factors can trigger the development and spread of malignant tumors, including tobacco use, obesity, alcohol consumption, infectious pathogens, exposure to ionizing radiation, and genetic factors, resulting in a complex pathophysiology and treatment. This pathology can be developed in various tissues and cell types, and among these, gastric cancer (GC) is the leading cause of cancer-induced deaths in Chile with an estimated mortality of 20/100,000 inhabitants (25.1/100,000 males and 13.2/100,000 females) for the last 10 years (Heise et al., 2009). Patients with early GC manifest symptoms that are indistinguishable from patients suffering from other benign diseases. These symptoms include anemia, dysphagia, or/and weight loss. Correspondingly, the majority of patients with advanced GC show symptoms including recurrent abdominal pain, anemia, weight loss, vomiting, and anorexia (Minsal, 2014). For this reason, it is desirable to develop therapeutic strategies to treat GC, as well as techniques that can predict risk of disease.

Nucleoside triphosphates such as adenosine-triphosphate (ATP) and uridine-triphosphate (UTP) are crucial for several metabolic processes and provide the energy necessary to carry all cellular processes. In addition to their crucial roles in cell metabolism, these molecules are also released into the extracellular space where they act as signaling molecules, activating various types of membrane receptors, which are termed purinergic receptors. These receptors include P2Y receptors (P2YRs), which are G-protein coupled (Abbracchio et al., 2006; Coddou et al., 2011; Jacobson et al., 2012) and P2X receptors (P2XRs), which are ATP-gated ionic channels that promote rapid depolarization as a consequence of cation influx through their cationic-selective pore (Coddou et al., 2011). Eight P2YR and seven P2XR subtypes have been identified (Abbracchio et al., 2006; Coddou et al., 2011; Jacobson et al., 2012). There is also a family of P1 G-protein coupled receptors that is activated by the purine nucleoside adenosine, the final product of ATP degradation by ectonucleotidases (Abbracchio et al., 2006; Jacobson et al., 2012).

Purinergic signaling is involved in several physiological and pathophysiological processes; ATP and other nucleotides can be released from most cell types in response to different stimuli and act as a paracrine or autocrine signal, or can be stored and released by vesicles which contain ATP together with other transmitters such as noradrenaline, neuropeptide Y, or acetylcholine in the peripheral nervous system, or with dopamine, gamma-aminobutyric acid (GABA) or glutamate in the central nervous system (Burnstock, 2013). Consistently, the actions of ATP as an extracellular signaling molecule can have short-term effects, for example in synaptic transmission, neuromodulation, and secretion, or long-term or trophic effects that include cell proliferation, differentiation, and death. Purinergic signaling and its receptors are present in several pathologies, including cancer, and there is a renewed effort to explore and develop therapies that affect purinergic signaling cascades. Nucleotides are abundant in the tumor microenvironment, especially ATP and adenosine, and this is in concordance with the proposed role of ATP as a danger signal and its participation in inflammatory processes (Di Virgilio, 2012). It has been shown in tumors that ATP accumulates in the extracellular space: the source of ATP can be either by leakage through damaged membranes or mediated by specific ATP transport across the plasma membrane. Moreover, recent studies have established that ATP is not only released by inflammatory-related processes, but also by processes directly related to cancer cell metabolism and antitumor immunity (Ghiringhelli et al., 2009). The final effect of extracellular ATP accumulation results in either increased or decreased tumor growth. The final response depends on several factors including the final concentration of ATP, degradation rate to adenosine, and the specific subtypes of purinergic receptors expressed in each particular type of tumor, and by its associated inflammatory cells (Di Virgilio, 2012). Purinergic signaling also controls the proliferation, growth, differentiation, and possibly metastasis of tumors due to its long-term or trophic effects. These effects are consequences of the activation of different signal transduction pathways that can lead to the generation of second messengers or activation of protein kinases or phosphatases which activate specific genes that are responsible for long-term effects (Burnstock and Verkhratsky, 2010). There is abundant evidence of the role of long-term purinergic signaling regulating proliferation. This evidence includes tumors from prostate, bladder, melanoma, and breast, among other organs (White and Burnstock, 2006). In most cases, but not all, P2YRs receptors are involved in proliferation-related events, while P2XRs mediate differentiation and cell death (White and Burnstock, 2006). In addition, purinergic signaling has been postulated to play an important role in the tumor microenvironment, participating in processes such as immunosuppression and in other normal/tumor cells interactions (Di Virgilio et al., 2018). High amounts of ATP are present in the tumor microenvironment and cancer cells have developed several strategies to use this ATP and the purinergic receptors involved to promote tumor growth and propagation, and to even suppress immune responses against cancer cells (Di Virgilio et al., 2018).

However, there is no information about the potential role of purinergic signaling in GC. A recent study from our group reported a significant increase in the expression of the P2Y_2_R in tumor biopsies from GC patients (Aquea et al., 2014). The results shown here not only indicate that there is a dramatic change in the expression profile of purinergic receptors in GC cells, but also suggest that purinergic signaling could be an important player in the pathophysiology of GC, reflected in the changes in proliferation of GC cells when purinergic signaling is pharmacologically manipulated. Thus, purinergic signaling could constitute a novel target for GC treatment.

## Materials and Methods

### Cell Culture

HEK293 and AGS cells were obtained from ATCC (Manassas, VA, United States); MKN-45 and MKN-74 cells were obtained from Riken Bank (Tsukuba, Ibaraki, Japan); GES-1 cells were kindly donated by Dr. Dawit Kidane (University of Texas at Austin, USA). Cell lines were maintained under regular cell culture conditions: Dulbecco’s modified Eagle’s medium (DMEM)-High-glucose for human embryonic kidney 293 cells (HEK293) and GES-1 (Gibco, NY, United States); F-12K for AGS cells (Corning, NY, United States); and RPMI 1640 for MKN-45 and MKN-74 cells (Corning, NY, United States). All cellular media was supplemented with 10% fetal bovine serum (Biological Industries, Kibbutz Beit-Haemek, Israel), 100 IU/mL penicillin G, and 100 μg/mL streptomycin sulfate (Sigma-Aldrich, St. Louis, MO, United States) and antibiotics. Cell lines were routinely subcultured after 4 days, and experiments were performed between the 8^th^ and 30^th^ passage. Transfection of AGS cells with P2X4R complementary DNA (cDNA) was conducted 24 h after plating the cells using 2 µg of deoxyribonucleic acid (DNA) and 5 µl of LipofectAMINE 2000 reagent (Invitrogen) in 2 ml of serum-free Opti-MEM. After 4.5 h of incubation, the transfection mixture was replaced with normal culture medium. Experiments were performed 24–48 h after transfection.

### Polymerase Chain Reaction

Total RNA was isolated from HEK293, AGS, and GES cells using TRIsure (Bioline, USA) following the manufacturer’s specifications. Briefly, cell samples were resuspended with 1 mL of TRIsure in RNase-free sterile tubes on ice and 200 µL of chloroform was added to each tube. The mixtures were vortexed for 30 s and centrifuged at 15,000 g for 15 min at 4°C. The aqueous phase was recovered and total RNA was precipitated with cold isopropanol (Merck, Darmstadt, Germany). Samples were centrifuged, and pellets were washed with 75% v/v ethanol–water diethyl pyrocarbonate (DEPC). Finally, RNA pellets were dried and dissolved in RNAse/DNAse free ultrapure water. Concentration was measured by ultraviolet–visible (UV-vis) spectrophotometry [Nanoquant Infinite M200 pro (TECAN, Männedorf, Switzerland)], and integrity was analyzed by electrophoresis on agarose gels. RNA (2 μg) was treated with RQ1 RNase-free DNase (Promega Corporation, Madison, WI, USA) in a final volume of 20 µl, and cDNA synthesis was performed using Moloney Murine Leukemia Virus (M-MLV) reverse transcriptase and oligo dT (Promega, Madison, WI, USA) according to the manufacturer’s protocol in a final volume of 40 µl.

Polymerase chain reactions (PCRs) were carried out using GoTaq Green Master Mix (Promega) in Stratagene Mx 3000p equipment (Agilent Technologies, Santa Clara, CA, USA). PCR primer sequences are listed in **Table 1**. Reactions were performed using 7.5 μL of GoTaq Green Master Mix, 0.5 μL of forward and reverse primer (10 μM), 1 μL of cDNA (200 ng), and 5.5 μL of ultrapure water (Gibco). Cycling conditions were: 95°C for 2 min, followed by 35 cycles consisting of 95°C for 35 s, 53–55°C for 35 s and 72°C for 1 min, with a final extension step of 72°C for 2 min. PCR product was confirmed on agarose gels with Gel Red (Biotium, Freemont, CA, USA).

**Table 1:** Primers for conventional polymerase chain reaction (PCR).

Gene	Sequence	Amplicon size
P2X1R	FP- 5´ -CCCACCATGGCACGGCGGT- 3´	767
	RP- 5´ -CCAACCACTCCACCCTTCTCA- 3´	
P2X2R	FP- 5´ -TCCACTTCTCCAAGGGCAAC- 3´	633
	RP- 5´ -AGTTGATAATGACCCCGATGAC- 3´	
P2X3R	FP- 5´ -TACGGGACACAGCCATTG- 3´	733
	RP- 5´ -CGGTACTCACTGCCATTT- 3´	
P2X4R	FP- 5´ -CACCCACAGCAACGGAGTCT- 3´	793
	RP- 5´ -TTTGATGGGGCTGTGGAGAG- 3´	
P2X5R	FP- 5´ -AACGTCTGTGCTGAGAATGAAG- 3´	280
	RP- 5´ -TCACATTGCTTTTGGAGAAGTTGA- 3´	
P2X6R	FP- 5´ -GGTCTATGTGGTAGGGTGGG- 3´	209
	RP- 5´ -GCGTCACAAGGAAGTTGGTC- 3´	
P2X7R	FP- 5´ -CTGCTGTCGCTCCCATATT- 3´	217
	RP- 5´ -CGCAGGTCTTGGGACTTCTT- 3´	
P2Y_1_R	FP- 5´ -CTGTATCAGCGTGCTGGTGT- 3´	517
	RP- 5´ -TTTCCTTGTGGCTCGGGAGA- 3´	
P2Y_2_R	FP- 5´ -TCCTGTTTCCCGCAGAGTTC- 3´	306
	RP- 5´ -CCATCCCAGGTGCCATTGAT- 3´	
P2Y_4_R	FP- 5´ -CGTGCCCAACCTGTTCTTTG- 3´	537
	RP- 5´ -CAGCTGCTATCCTCAGGCAG- 3´	
P2Y_6_R	FP- 5´ -ACACTCCTGATATGTCTCTCGG- 3´	616
	RP- 5´ -GCATAGAAGAGGAAGCGGACC- 3´	
P2Y_11_R	FP- 5´ -CATCACCTGCATCAGCCTCA- 3´	195
	RP- 5´ -CTGGCCACGCTGCAGTT- 3´	
P2Y_12_R	FP- 5´ -TTACACCCTGAGCCAAACCC- 3´	184
	RP- 5´ -TGCAGAATTGGGGCACTTCA- 3´	
P2Y_13_R	FP- 5´ -TCCTAATGCTTGTGTTTTATGTGGT- 3´	160
	RP- 5´ -CTGGCAAAATGAAATGGAGCA- 3´	
P2Y_14_R	FP- 5´ -TGCCAGAATCCCCTACACAA- 3´	163
	RP- 5´ -GATTTCCCTAAACGGCTGGC- 3´	

### Real-Time qPCR

Analysis by real-time quantitative PCR (RT-qPCR) was performed to compare the level of gene expression of P2Y_2_R and P2X4R receptor from gastric mucosa from patients diagnosed with GC, obtained from the Hospital San Pablo, Coquimbo. Biopsies from both tumoral and adjacent healthy mucosa were obtained from each patient, and protocols were approved by the Ethical-Scientific Committee of the Faculty of Medicine of the Universidad Católica del Norte (CECFAMED-UCN). Patients voluntarily signed the respective informed consent form. Additionally we performed RT-qPCR analyses for P2X4R, P2X7R, P2Y_1_R, P2Y_2_R, and P2Y_4_R for the GC-derived cell lines AGS, MKN-45, and MKN-74 and compared expression levels to the gastric mucosa-derived cell line GES-1. Total RNA was extracted using RNeasy Mini Kit (Qiagen), and each experiment was performed in triplicate. First-strand cDNA was synthesized from 500 ng of total RNA with Affinity Script QPCR cDNA Synthesis Kit (Agilent Technologies). Gene-specific primers for purinergic receptors were designed using Primer 3 software (http://bioinfo.ut.ee/primer3-0.4.0/) to have melting temperatures of 60°C and to generate PCR products of approximately 100–200 bp. β2-Microglobulin gene (B2M) was used as an endogenous control to normalize experimental results. We chose B2M as our housekeeping gene because although variations in B2M have been observed in other cancer types, in our GC biopsies their expression has not been affected like in other housekeeping genes such as β-actin (Aquea et al., 2014). Information about the primer sequences is shown in **Table 2**. Primers were designed using Primer-Blast software from National Center for Biotechnology Information (NCBI), and efficiency was calculated from the slope of each standard curve, finding efficiency values between 90% and 101% obtained for all genes evaluated. Each qPCR contained 12.5 µL of 2× Brilliant II SYBR Green QPCR Master Mix (Agilent Technologies), 10 ng cDNA, and 0.45 µM (final concentration) of each primer, in a final volume of 25 µl. RT-qPCR reactions were run at the Applied Biosystems StepOne™ (Applied Biosystems). QPCR conditions were as follows: initial denaturing for 10 min at 95°C, followed by 35 cycles of 10 s at 95°C, 45 s at 60°C, 60 s at 95°C, followed by 30 s at 60°C and 30 s at 95°C. The comparative 2(-ΔΔCT) method was used to quantify the relative abundance of transcripts (Livak and Schmittgen, 2001).

**Table 2:** Primers for real-time quantitative PCR (qPCR).

Gene	Sequence	Amplicon size
P2X4R	FP- 5´ -ACTATGATCAACATCGGCTCTG- 3´	149
	RP- 5´ -CTAGCAAGACCCTGCTCGTAAT- 3´	
P2X7R	FP- 5´ -CCAGTCACCAAACATGAGAGAG- 3´	180
	RP- 5´ -GTTCCAGTTACCTGTTGCTGTG- 3´	
P2Y_1_R	FP- 5´ -TCGGTTACTGCTGCTTAGTTCTAC- 3´	118
	RP- 5´ -TGAACTCGCTGGTAGTGTGTTAC- 3´	
P2Y_2_R	FP- 5´ -CTGGTAGCGAGAACACTAAGGAC- 3´	190
	RP- 5´ -TTTGATGGGGCTGTGGAGAG- 3´	
P2Y_4_R	FP- 5´ -TTCATCTTCCGCCTCCGACC- 3´	89
	RP- 5´ -GGCAGCGACAGCACATACAA- 3´	
B2M	FP- 5´ -AGATGAGTATGCCTGCCGTG- 3´	120
	RP- 5´ -TCATCCAATCCAAATGCGGC- 3´	

### Protein Extraction and Western Blot Analysis

AGS or GES-1 cells were washed twice with phosphate-buffered saline (PBS) at 4°C and lysis buffer containing 50 mM Tris-HCl (pH 7.5), 0.1 M NaCl, 50 mM MgCl_2_, and 1% Triton X-100 with complete protease inhibitors (Thermo Scientific^™^, Waltham, MA, United States). Protein concentrations were determined using a bicinchoninic acid (BCA) protein assay (Thermo Scientific^™^). Equal amounts of total protein were loaded and separated by sodium dodecyl sulfate–polyacrylamide gel electrophoresis (SDS-PAGE) gel electrophoresis, and then were transferred to polyvinylidene fluoride (PVDF) membranes (Thermo Scientific^™^). Membranes were blocked with buffer [5% nonfat dry milk in Tris-buffered saline (TBS) with 0.05% Tween-20 (TBS-T)] at room temperature for 1 h. Blots were incubated with primary antibodies against P2X4R, P2X7R, P2Y_2_R, P2Y_1_R (Alomone Labs, Jerusalem, Israel), and β-actin (Sigma-Aldrich, St. Louis, MO, USA) at 4°C overnight. The blots were washed with TBS-T five times and incubated for 1 h at room temperature with corresponding secondary antibodies diluted in 1% nonfat dry milk blocking buffer. The immunoreactive bands were visualized by enhanced chemiluminescence detection using SuperSignal West Pico (Thermo Scientific^™^) and imaged with a C-DiGit scanner.

### Immunofluorescence Microscopy

Human AGS, GES-1, MKN-45, and MKN75 cells were cultured on glass coverslips in 24-well dishes in their respective culture medium at 37°C (as described above). After cells reached 70–90% confluence, the cells were washed three times with phosphate-buffered saline containing 0.1 mM CaCl_2_ and 1 mM MgCl_2_ (PBS-CM), fixed for 20 min at room temperature with 3.7% paraformaldehyde, and quenched for 10 min with 50 mM NH_4_Cl. Cells were permeabilized with 0.2% Triton X-100 in PBS-CM for 10 min at room temperature (RT) and blocked with PBS–1% bis(trimethylsilyl)acetamide (BSA) for 30 min. Then cells were incubated with primary antibodies against P2X4R, P2X7R, or P2Y_2_R (1:100) (Alomone Labs, Jerusalem, Israel) in a humidified chamber overnight at 4°C. Following incubation, cells were washed three times with PBS and incubated with respective secondary antibodies, Alexa Fluor 488-conjugated goat anti-mouse (or anti-rabbit) antibody (1:500) (Thermofisher Scientific^™^, Waltham, MA), together with nuclei stain Hoechst 33342 (1:2,000) (Sigma-Aldrich) for 1 h at RT. After washing with PBS, the coverslips were mounted with Prolong (Thermofisher Scientific^™^, Waltham, MA). Single focal images were taken using Zeiss LSM 800 confocal microscope (Carl Zeiss, Heidelberg, Germany) plan-apochromat 63x/1.46 oil objective. All images for each antibody were acquired under identical condition settings: 16-bit, 1,024 × 1,024 pixels, avoiding signal saturation, pinhole adjusted to 1 Airy unit, gain between 650 and 700 V and laser power ranging from 0.2 to 0.8%. The acquired images were processed generating regions of interest (ROIs) using Zen image software, and figure composition was performed using Adobe Photoshop (CS, Adobe Systems).

### Intracellular Calcium Measurements

Cultured AGS or GES-1 cells at 70–85% confluence were loaded with Fluo4-AM (3 µM in dimethyl sulfoxide (DMSO), Molecular Probes, Eugene, OR) for 30 min at 37°C. Cells were washed twice with PBS and incubated for 30 min at 37°C and then mounted in a perfusion chamber that was placed on the stage of a confocal microscope (Zeiss LSM800) at 22–24°C. Purinergic agonists [ATP, UTP, benzoylbenzoyl ATP (BzATP), MRS2768, or α,β methylene ATP (α,β-meATP)] were added at the corresponding time at a final concentration of 100 µM in a bath solution. Cultured cells were briefly illuminated adjusting laser power (≤0.5%) and scan speed (6–7), using the ZEN 2.1 software (Zeiss). ROIs were simultaneously selected on cell somata containing Fuo4 fluorescence (laser excitation 480 nm, emission 510 nm) in a field with more than 150 cells. Images were collected at 2-s intervals during a continuous 12–15 min period. Imaging was carried out in 16-bit (0–65,000 units of fluorescence scale) with highly sensitive GaAsP detector (Zeiss). The Ca^2+^ transients, defined by an amplitude greater than two times the noise level, were acquired and analyzed offline using ZEN blue 2.1 software.

### Cell Proliferation Studies

Studies were performed using a Vybrant^®^ 3-(4,5-dimethylthiazol-2-yl)-2,5-diphenyltetrazolium bromide (MTT) Cell Proliferation Assay Kit (Thermo Scientific™, Waltham, MA) in AGS, MKN-45, MKN-74, and GES-1 cells. The MTT assay demonstrates several changes in mitochondrial activity, which are measured by the enzymatic reduction of MTT to formazan (Mosmann, 1983). Measuring the reductive capacity of mitochondria is widely used as evidence of cell metabolic functionality because formazan production is associated with several enzymes including peroxidases, oxidases, and dehydrogenases, which are essential for sustaining life. Therefore, cell proliferation assays are one of the main approaches for assessing cell viability (Prabst et al., 2017). Cells were incubated with 5,000 cells/well in 96-well plates for 24 h. After this period, conditions were changed by adding purinergic agonists ATP, UTP, ADP (Sigma-Aldrich, St. Louis, MO), MRS2768 (Tocris, Minneapolis, MN) and/or antagonists suramin, pyridoxal phosphate-6-azo(benzene-2,4-disulfonic acid) (PPADS), AR-C 118925XX, BX430, and AZ10606120 dihydrochloride (Tocris, Minneapolis, MN). After an additional 24 h, proliferation was measured by MTT according the provider’s protocol. In brief, culture media was replaced with an MTT-containing solution. After a 4-h incubation, 50 µL DMSO was added to the cells and mixed with a pipette. This mix was incubated for 10 min, and absorbance was measured at 540 nm in a NOVOSTAR multiplate reader. Cell viability was determined as a parameter of proliferation using the formula: % cell viability = (OD treatment/OD control) × 100.

### Data Analysis

Data was presented as mean ± standard error of at least three different experiments using GraphPad Prism 5.01 software (GraphPad, San Diego, CA). Statistical analyses included nonparametric Mann–Whitney tests and Kruskall–Wallis test followed by the Dunn’s multiple comparison test using GraphPad software. Statistical significance was set when *p* < 0.05.

## Results

### Expression of Purinergic Receptors in Tumoral and Nontumoral Cell Lines

**Figure 1:**
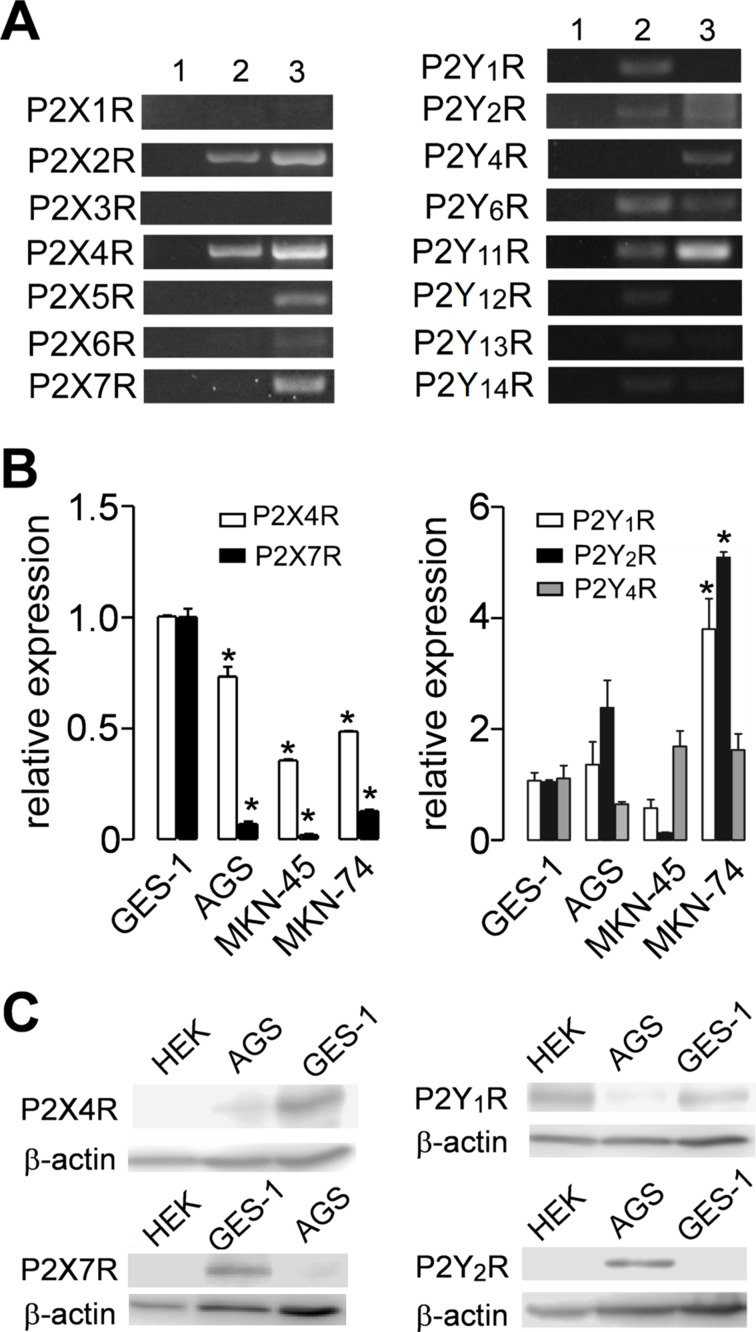
Purinergic receptor pattern expression in normal and cancer-derived cell lines. **(A)** Representative polymerase chain reaction (PCR) from total RNA obtained from AGS (2) or GES-1 (3) cells for P2X receptors (P2XRs) (left) and P2Y receptors (P2YRs) (right). Lane 1 represents a blank control; the data shown is representative of at least three separate experiments. **(B)** Summary of quantitative PCR (qPCR) experiments showing RNA levels of P2X4R and P2X7R (left graph) or P2Y_1_R, P2Y_2_R, and P2Y4R (right graph) in control GES-1 cells or in gastric cancer (GC)-derived AGS, MKN-45, or MKN-74 cells. *p < 0.05, Kruskal–Wallis and Dunn´s tests. **(C)** Western blot analysis for the expression of P2X4R, P2X7R, P2Y_1_R, P2Y_2_R, and β-actin in HEK293, AGS, and GES-1 cells. Blots are representative of three different experiments.

**Figure 2:**
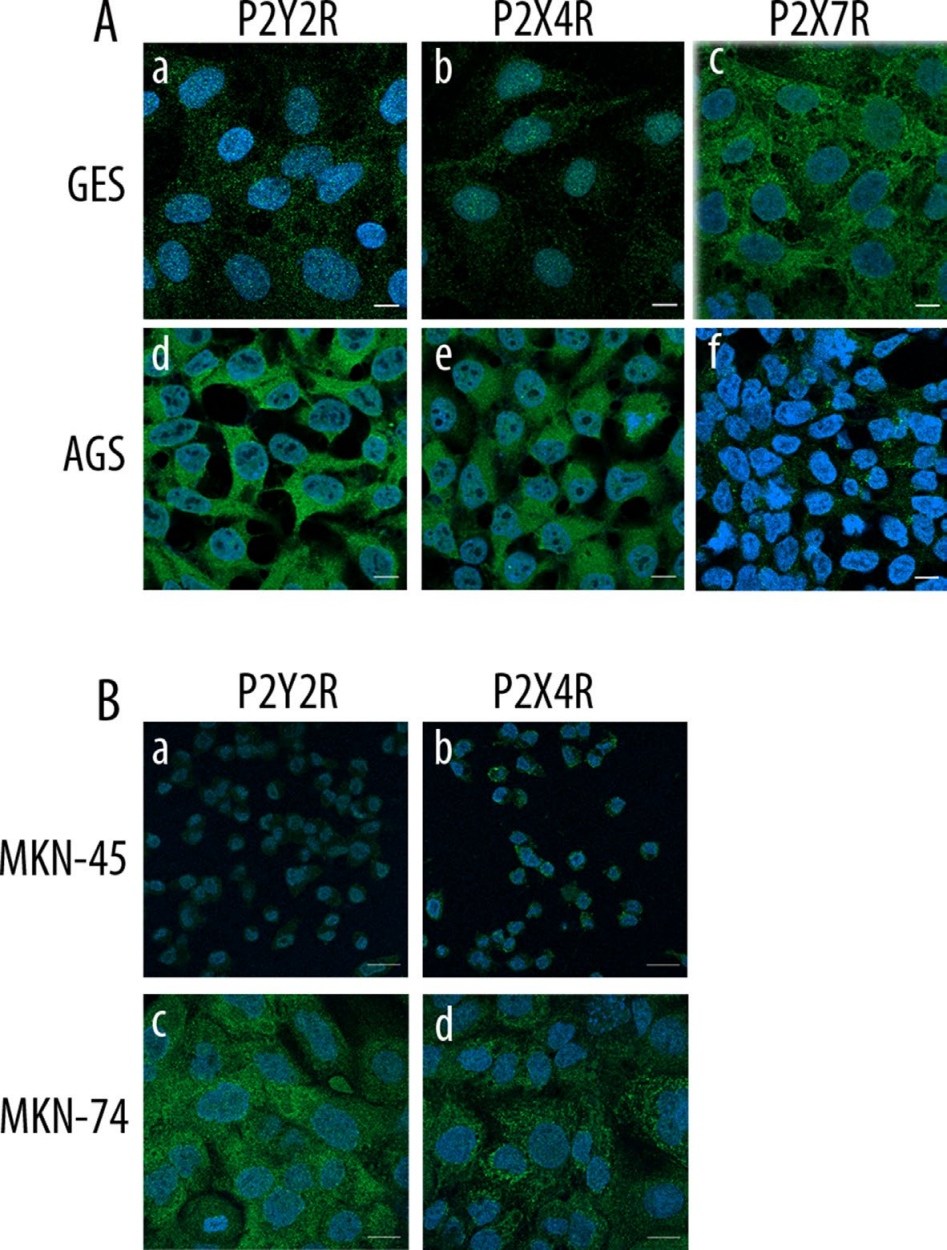
Protein expression of purinergic receptors in normal and cancer-derived cell lines. **(A)** Confocal images from GES-1 and AGS cells showing immunofluorescence for P2Y_2_R, P2X4R, and P2X7R; scale bar 10 µm. **(B) **Confocal images from MKN-45 and MKN-74 cells showing immunofluorescence for P2Y_2_R and P2X4R; scale bar 10 µm. In all cases the images shown are representative of three different samples.

We assessed the expression of purinergic receptors in AGS, a cell line derived from a gastric adenocarcinoma, and GES-1, a cell line derived from normal gastric epithelium cells. In a first approach, we used PCR to evaluate the expression of several purinergic receptors. We found that both cell lines express several purinergic receptors; with the most remarkable difference being that GES-1 cells express more P2X receptors subtypes than AGS cells, which in general express more P2Y receptors (**Figure 1A**). The main purinergic receptors expressed by AGS cells were P2X2R, P2X4R, P2Y_1_R, P2Y_2_R, P2Y_6_R, P2Y_11_R, and P2Y_12_R. Meanwhile, GES-1 cells expressed P2X2R, P2X4R, P2X5R, P2X7R, P2Y_2_R, P2Y_4_R, P2Y_6_R, and P2Y_11_R (**Figure 1A**). Next, we compared the RNA levels of some P2XRs and P2YRs by qPCR to compare the changes between GES-1 cells, derived from healthy gastric mucosa and the GC cell lines AGS, MKN-45, and MKN-74. **Figure 1B** shows the decrease in expression of P2X4R and P2X7R in GC cell lines, as compared to GES-1 cells. In the case of the P2Y_2_R, we found an increase in the expression in AGS and MKN-74 cells, but not in MKN-45, whereas the P2Y_1_R expression was increased in MKN-74 cells, as compared to GES-1 cells (**Figure 1B**). However, levels of P2Y_4_R RNA were relatively similar in all cell lines tested (**Figure 1B**). Then, we directly measured protein expression by Western blot, showing the presence of P2Y_1_R, P2Y_2_R, and P2X4R, and the absence of P2X7 in AGS cells (**Figure 1C**). Interestingly, in GES-1 cells, expression of P2X4R was strongly increased when cells were allowed to form a confluent monolayer, resembling the “normal” state of epithelium, whereas the expression of the other purinergic receptors was not affected by cell confluence (**Supplemental Figure 1**). To confirm these results, we performed immunofluorescence experiments as shown in **Figure 2**; these experiments confirmed the expression of the P2Y_2_R and the P2X4R in GES-1 and AGS cells; and the expression of P2X7R in GES-1 but not in AGS cells (**Figure 2A**). In addition, we confirmed a strong expression of P2Y_2_R and P2X4R in MKN-74 cells as compared to MKN-45 cells that exhibited a very low expression of both receptors (**Figure 2B**). Altogether, these experiments confirmed the expression of purinergic receptors in tumor-derived and healthy mucosa-derived gastric cell lines.

### Purinergic Agonists Induce Intracellular Calcium Increases

**Figure 3:**
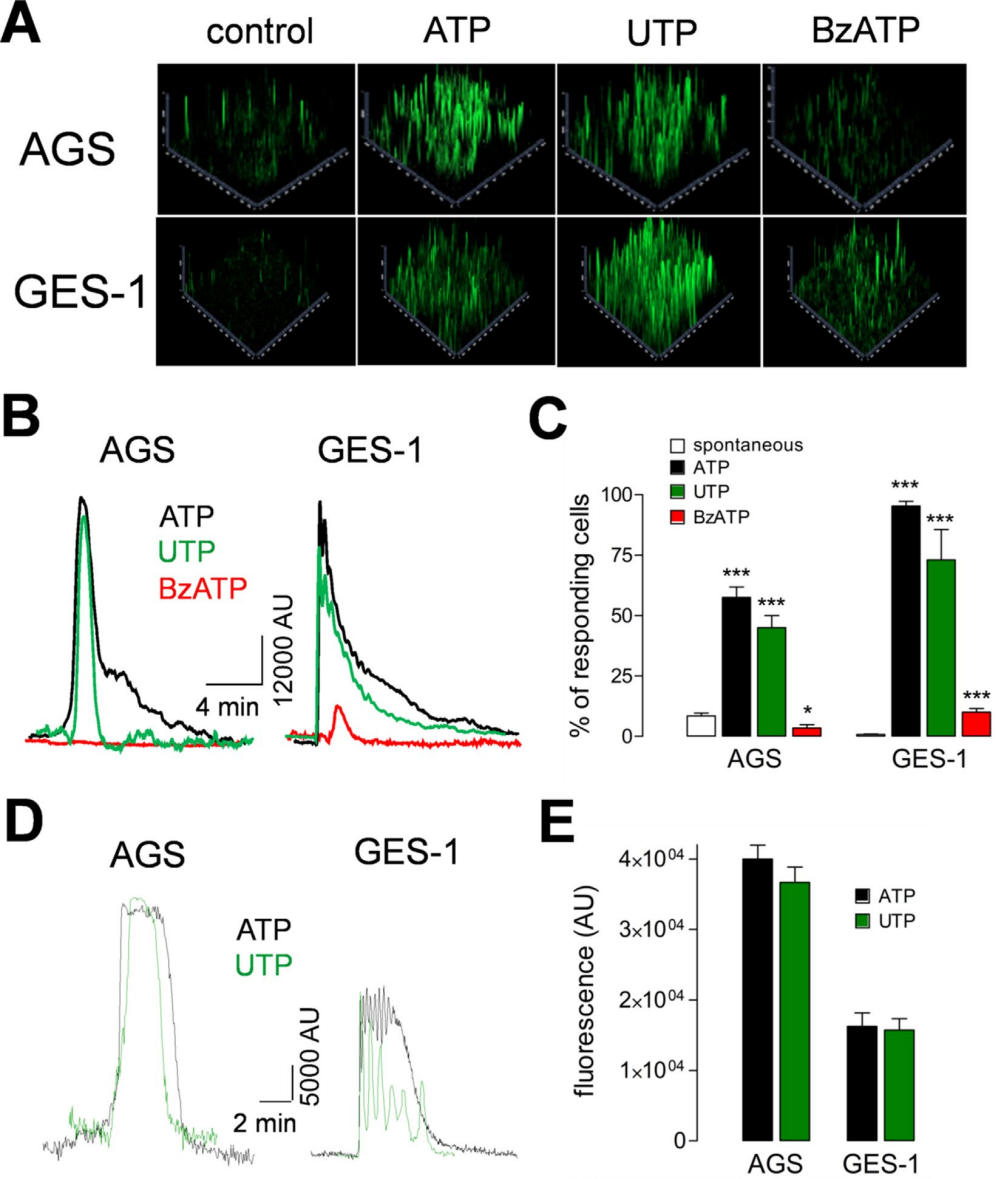
Purinergic receptor induced increases of intracellular calcium. **(A)** Representative 2.5D representation of ATP, UTP, or BzATP (100 μM) induced increases of [Ca^2+^]_i_ in AGS and GES-1 cells. **(B)** Total increase of [Ca^2+^]_i_ induced by ATP (black traces), UTP (green traces), and BzATP (red traces) in AGS and GES-1 cells, in four to six fields. **(C)** Percentage of responding cells for spontaneous activity (open bars), ATP (black bars), UTP (green bars), and BzATP (red bars). *p < 0.05; ***p < 0.001, ANOVA test, *n* = 4–11. **(D)** Representative recordings from single AGS or GES-1 cells showing the increase of [Ca^2+^]_i_ induced by 100 µM ATP (black traces) or UTP (green traces). **(E)** Summary of the maximal relative fluorescence, measured in arbitrary units (AU) induced by ATP (black bars) or UTP (green bars) in AGS and GES-1 cells, *n* = 21–24.

**Figure 4:**
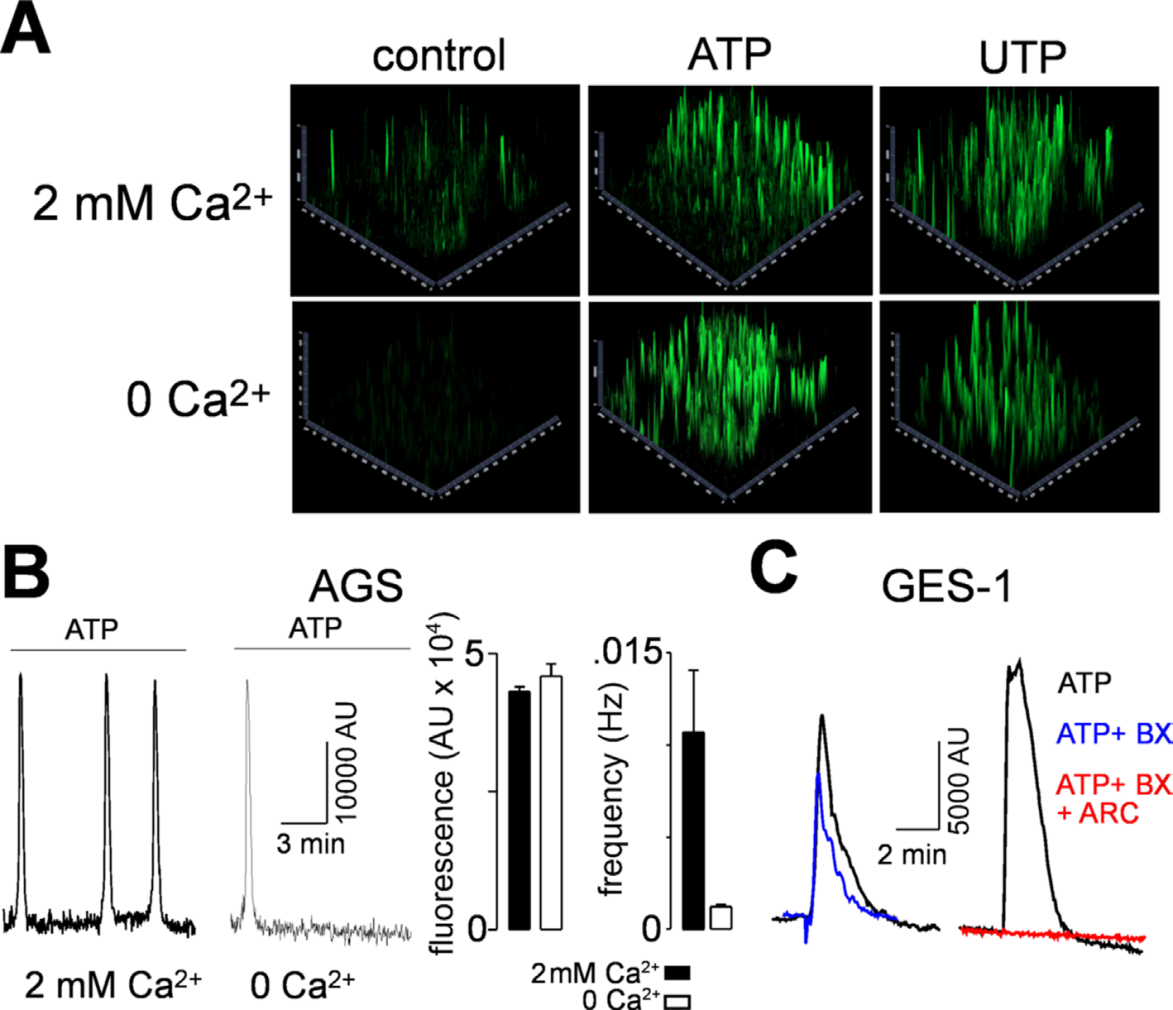
Contribution of P2YRs and P2XRs to increases in [Ca^2+^]_i_. **(A)** 2.5D representations of AGS cells showing spontaneous (control) activity after the application of 100 µM ATP in the presence (upper images) or absence (lower images) of extracellular calcium. **(B)** Left, representative recordings from AGS cells showing the 100 µM ATP-induced spikes in the presence or in the absence of extracellular calcium. Right, summary of the maximal relative fluorescence and frequency of the ATP-induced spikes in the presence (black bars) or absence (open bars) of 2 mM extracellular Ca^2+^. **(C) **Representative recordings showing [Ca^2+^]_i_ increases induced by 100 µM ATP alone (black traces) or together with BX430 (blue traces) or BX430 and ARC-118925XX (red trace).

In order to characterize the functional responses of purinergic receptors, we measured the increase of intracellular calcium ([Ca^2+^]_i_) in AGS and GES-1 cells evoked by purinergic agonists. Both ATP and UTP elicited robust increases of [Ca^2+^]_i_ in both cell lines. **Figure 3A** shows 2.5D images of a group of AGS and GES-1 cells and the responses evoked by 100 µM ATP, UTP, or BzATP. The percentage of responding AGS cells was 57 ± 4% and 48 ± 6% for ATP and UTP, respectively, whereas in GES-1 the percentage of responding cells was 95 ± 2% and 73 ± 12% for the same agonists (**Figure 3C**). We compared the total response, evoked by purinergic agonists we found that the effects of ATP and UTP were very similar in amplitude, with the difference that ATP induced longer-lasting responses as compared to UTP in both cell lines (**Figure 3B**). When we analyzed individual cell responses we found that ATP and UTP evoked responses with comparable relative amplitudes for the first peak of each spike (**Figure 3D and E**); however, the maximum fluorescence of these peaks was higher in AGS as compared to GES-1 cells (**Figure 3E**). In addition, we found a small but significant spontaneous activity in AGS but not in GES-1 cells (**Figure 3A and C**); this activity disappeared after removing calcium from the recording extracellular solution (**Figures 4A and B**). Interestingly the amplitudes of ATP and UTP evoked very similar responses in AGS cells in the presence or absence of extracellular calcium. However, the number of events was approximately one spike every 5 min in the presence of ATP and calcium, and was reduced to a single spike when extracellular calcium was removed (**Figure 4B**). To test the specific contribution of P2Y_2_R in AGS cells we used the specific agonist MRS2768, which evoked robust increases in [Ca^2+^]_i_ (**Supplemental Figure 2A**). Other purinergic agonists like α,β-meATP (P2X1 and P2X3 preferring) or BzATP (P2X7 preferring) did not elicit significant effects (**Supplemental Figures 2B** and **C**). These results strongly suggest that these experiments are mainly reflecting the activity of the P2Y_2_R and do not reflect potential contributions of P2XRs, such as P2X4R. To corroborate this, we performed recordings in GES-1 cells stimulated with only 100 µM ATP, or preincubated with the P2X4R specific antagonist BX430 (Ase et al., 2015); alone or with the P2Y_2_R-antagoinist AR-C118925XX. Whereas the application of BX430 has a modest inhibitory effect on the ATP induced increase of [Ca^2+^]_i_,the joint application of both BX430 and AR-C118925XX completely inhibited the increase of [Ca^2+^]_i_,induced by ATP (**Figure 4C**).

### Proliferation Studies in Cell Lines

**Figure 5:**
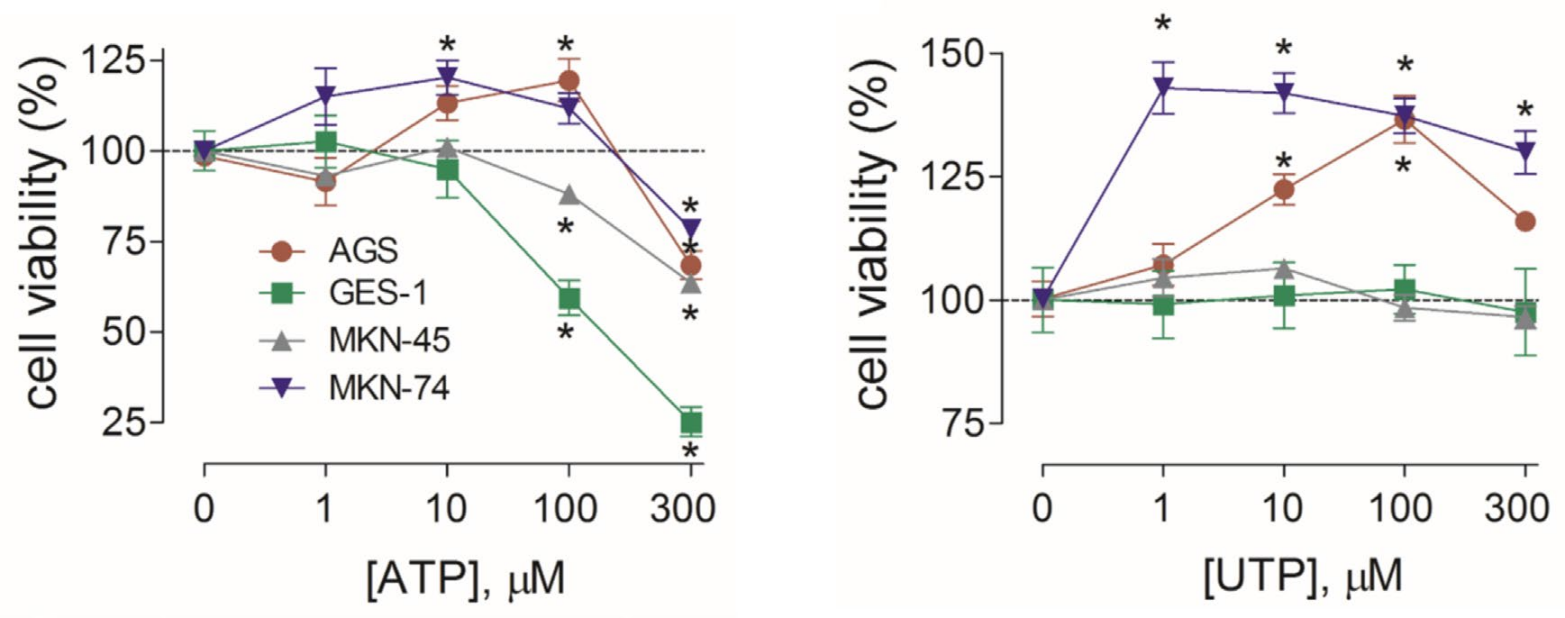
Summary of the effects of ATP and UTP in the proliferation of gastric cell lines. Changes in cell proliferation assessed by cell viability in the absence or presence of 1–300 µM ATP (left graph) or UTP (right graph) for the AGS (red), GES-1 (green), MKN-45 (gray), or MKN-74 (purple) cells. *p < 0.05, Kruskal–Wallis and Dunn’s tests, *n* = 4–6.

**Figure 6:**
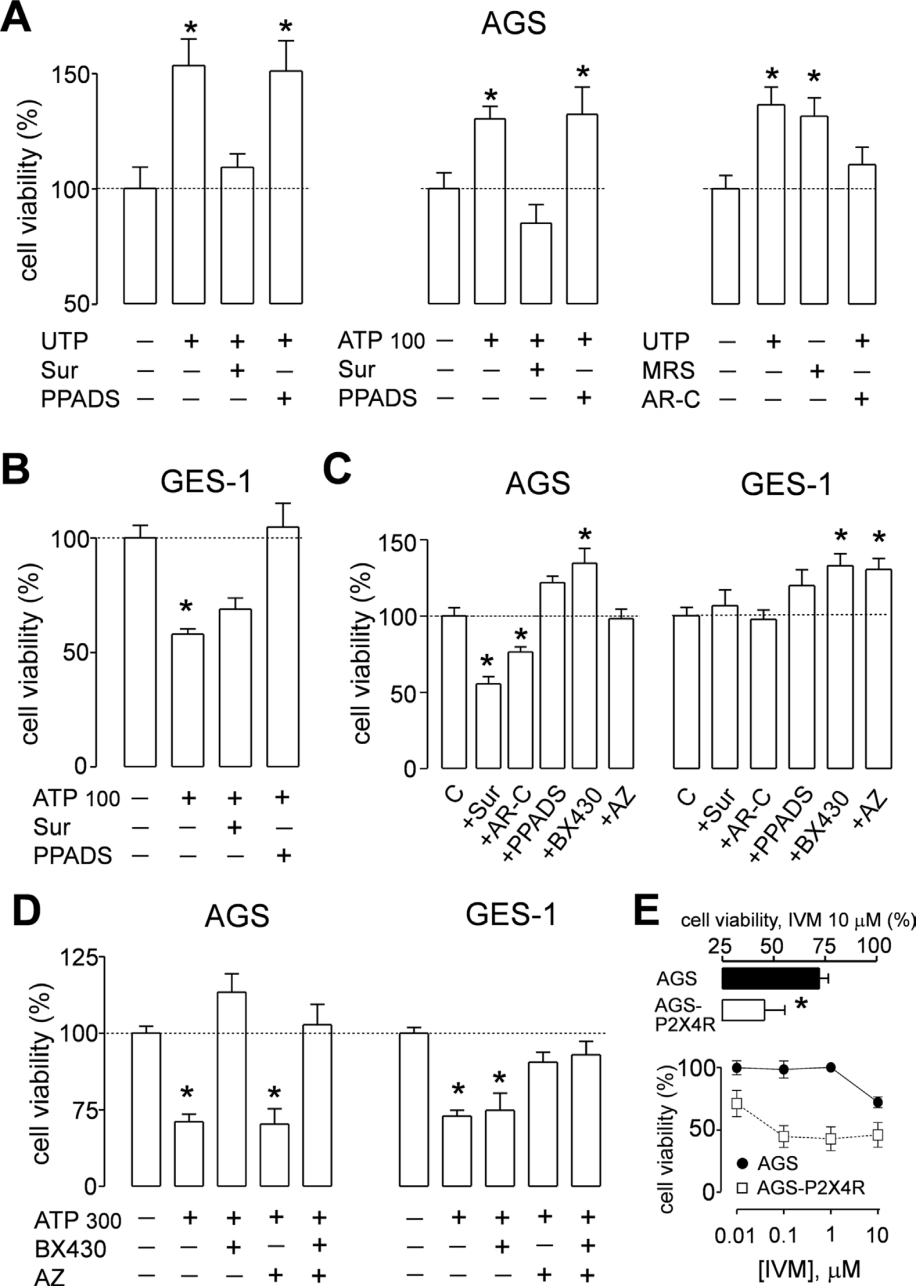
Effects of purinergic agonists and antagonist on the proliferation of AGS and GES-1 cells. **(A)** Summary of cell viability experiments on AGS cells showing the effect of: *left graph*, 100 µM UTP alone, or coapplied with 100 µM suramin (Sur) or 100 µM PPADS; *middle graph*, 100 µM ATP alone, or coapplied with 100 µM suramin (Sur) or 100 µM pyridoxal phospate-6-azo(benzene-2,4-disulfonic acid) (PPADS); *right graph*, effects of 100 µM UTP alone, 10 µM MRS2768 alone, or 100 µM UTP plus 10 µM ARC-118925XX, *n* = 4–7. **(B)** Summary of cell viability experiments in GES-1 cells showing the effect of 100 µM ATP alone, and coapplied with 100 µM suramin (Sur) or 100 µM PPADS, *n* = 3–5. **(C)** Summary of the effects of purinergic antagonists alone on cell viability applied to AGS (left) and GES-1 cells (right). The antagonists used were suramin (Sur) (100 µM), PPADS (100 µM), ARC-118925XX (AR-C) (10 µM), BX430 (10 µM), and AZ10606120 (AZ) (3 µM), *n* = 4–6. **(D)** Summary of cell viability experiments on AGS (left) and GES-1 (right) cells showing the effect of 300 µM ATP alone, and coapplied with 10 µM BX430 or 3 µM AZ10606120 (AZ), *n* = 3–4. **(E)** Effects of ivermectin (IVM) in AGS cell proliferation; *lower graph*: concentration–response curve for 1 nm–10 µM IVM and its effect on cell viability on control (closed circles) or P2X4R-overexpressing (open squares) AGS cells; *upper graph*: detail of cell viability in control (black bar) or P2X4R-overexpressing (open bar) AGS cells treated with 10 µM IVM, *n* = 3–4 in all cases *p < 0.05, Kruskal–Wallis and Dunn´s tests.

To determine whether activation of purinergic receptors has consequences in cell proliferation of GC-derived and healthy gastric cell lines, we performed MTT cell viability assays. We first evaluated the effects of ATP, the endogenous ligand of purinergic receptors, and measured the changes in cell proliferation. In AGS and MKN-74 cells, ATP induced a biphasic response; whereas 1–100 µM increased cell viability, 300 µM ATP caused a 25% decrease in cell viability, as compared to their respective untreated control cells (**Figure 5A**). On the other hand, MKN-45 and GES-1 cells were only inhibited by 10–300 µM ATP in a concentration-dependent manner (**Figure 5A**). In another set of experiments, we evaluated the effects of UTP, a specific agonist for P2Y_2_R and P2Y_4_R. With this agonist no inhibition was observed, only an increase of cell viability at 1–300 µM UTP in AGS and MKN-74 cells (**Figure 5B**). In the case of MKN-45 and GES-1 cells, no effect of UTP was observed at the concentrations tested (**Figure 5B**). We also tested the effects of ADP, a selective agonist for P2Y_1_R and P2Y_11_R, finding no significant changes in AGS cell proliferation (**Supplemental Figure 3**). These results not only confirmed that different receptor subtypes are mediating the purinergic responses, but also suggest that these receptors could have different roles in cell proliferation. To test this, we performed additional experiments using purinergic agonists and antagonists and evaluated their effects on AGS and GES-1 cells proliferation. **Figure 6** shows the conclusion of these experiments. In the first series of experiments, we used the P2Y_2_R and P2Y_4_R agonist UTP. The increase in cell proliferation induced by 100 µM UTP was significantly prevented by the wide range antagonist suramin but not by the P2X-preferring PPADS (**Figure 6A**, left graph). Suramin also inhibited the increase in cell proliferation induced by 100 µM ATP, and interestingly after this treatment cell viability decreased, as compared to untreated control cells; PPADS did not significantly affect AGS cell proliferation induced by ATP (**Figure 6A**, middle graph). We confirmed the pivotal role of the P2Y_2_R in cell proliferation using the specific agonist MRS2768, which increased AGS cell viability similarly to UTP (**Figure 6A**, right graph) and the specific P2Y_2_R antagonist AR-C118925XX which prevented the UTP increase in cell viability (**Figure 6A**, right graph). On the other hand, in GES-1 cells, the decrease in cell viability induced by ATP was prevented by wide range P2X antagonist PPADS but not by suramin, which does not inhibit P2X4R or P2X7R (**Figure 6B**). Seeing as ATP and related nucleotides have been postulated as paracrine and/or autocrine signaling molecules, we evaluated the effect of applying purinergic antagonists to control cells (with no exogenous ATP). In these experiments, AGS cell viability was significantly decreased with suramin and AR-C118925XX (**Figure 6C**) but was increased by PPADS and by the P2X4R-specific antagonist BX430, whereas the P2X7R antagonist AZ10606120 had no effect (**Figure 6C**). In GES-1 cells, P2Y-preferring antagonists did not induce any effect on basal cell proliferation and only the application of BX430 induced a significant increase in cell viability (**Figure 6C**). Contrary to what was observed in AGS cells, AZ10606120 also increased basal proliferation in GES-1 cells (**Figure 6C**). Altogether, our results indicate that the P2X4R, not P2X7R, is the purinergic receptor that decreases cell proliferation in GC-derived cell lines. To confirm this, we performed additional experiments using a high ATP concentration (300 µM), which decreases cell proliferation. This decrease was completely inhibited in AGS cells when ATP was coapplied with the P2X4R antagonist BX430, but not with the P2X7R antagonist AZ10606120 (**Figure 6D**, left). However, in GES-1 cells AZ10606120 prevented the decrease in cell viability induced by 300 µM ATP (**Figure 6D**, right). Since P2X4R plays an important role mediating the protective effects of nucleotides, we tested the effect of the positive allosteric modulator ivermectin (Khakh et al., 1999), finding that the isolated application of 10 µM ivermectin was able to decrease basal proliferation of AGS cells, and when P2X4R was overexpressed in these cells the effects of IVM were more pronounced and started at 0.1 µM (**Figure 6E**).

### Changes in the Expression of P2Y_2_ and P2X4 Receptors in Gastric Cancer-Derived Biopsies and Gastric Cell Lines

**Figure 7:**
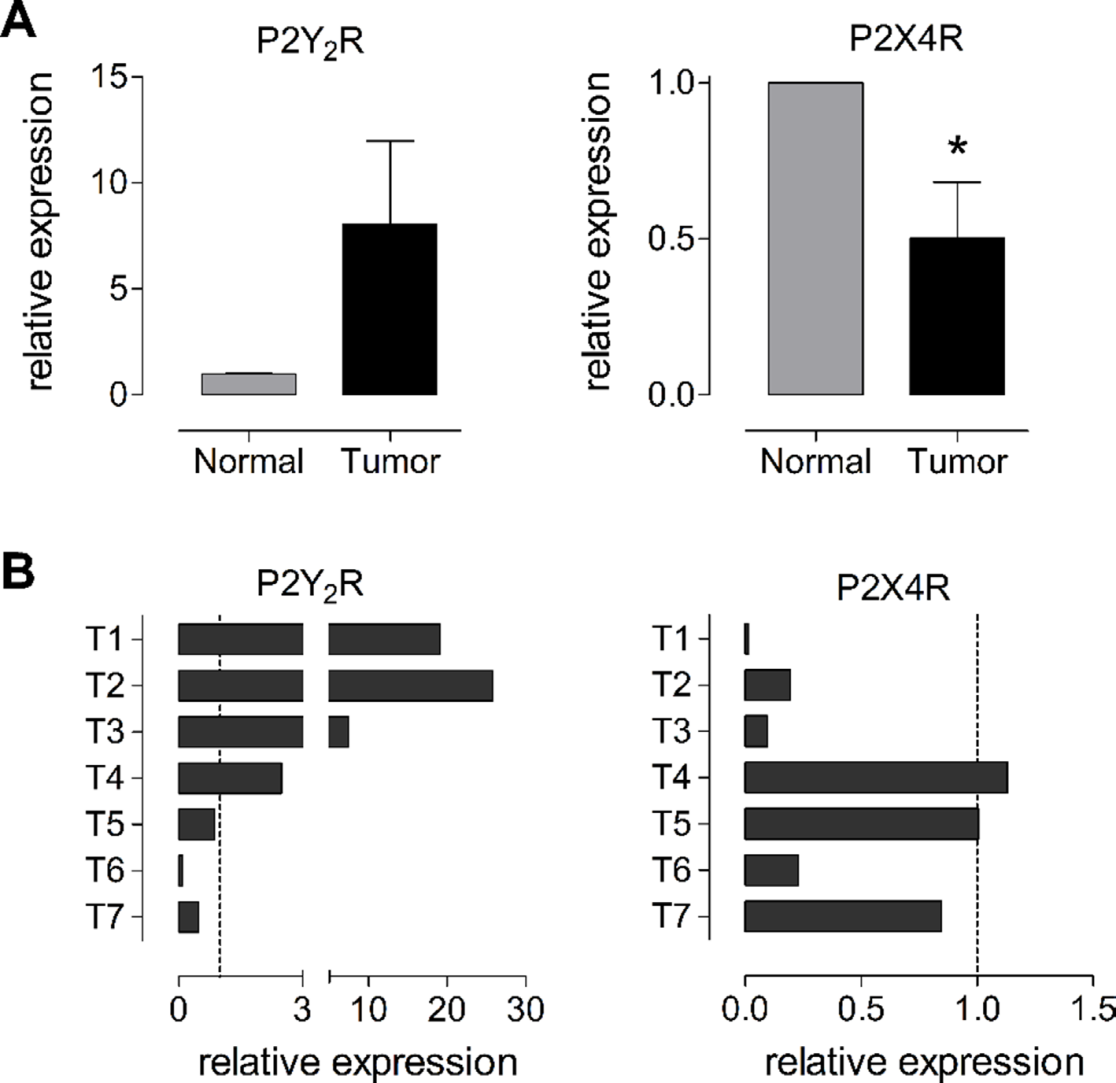
Changes in RNA levels of P2Y_2_R and P2X4R in human biopsies from normal and tumoral gastric tissues. **(A) **Summary of the changes of RNA measured by qPCR for P2Y_2_R (left graph) or P2X4R (right graph) taken from seven different patients diagnosed with GC. Changes in RNA levels were measured comparing the expression of the purinergic receptor in the tumor with its levels in the adjacent healthy gastric mucosa. *p < 0.05, paired *t*-test. **(B)** Individual changes in P2Y_2_R (left) and P2X4R (right) RNA levels in the seven different tumor biopsies analyzed. Dotted line represents the basal expression in adjacent healthy gastric mucosa.

We evaluated the changes in P2Y_2_R and P2X4R expression in tumors from patients diagnosed with GC and compared the results to their respective adjacent nontumoral gastric mucosa by qPCR. We analyzed seven GC tumors and their respective healthy gastric and we found a high degree of variation between the different tumors analyzed. The tumors were in both early and advanced adenocarcinomas, from five males and two females, with no metastasis. In general, P2Y_2_R RNA was increased 8.0 ± 3.9 fold in tumors as compared to normal tissue, whereas the P2X4R RNA decreased by half, 0.5 ± 0.2 fold (**Figure 7A**). However, as mentioned above, we found a high degree of variation between the different samples; in the case of P2Y_2_R we found a high (more than 10-fold) overexpression in T1, T2, and T3 and an important decrease in expression in T6 and T7 (**Figure 7B**). For P2X4R we found an important decrease of expression in T1, T2, T3, and T6 and no significant changes in T4, T5, and T7 (**Figure 7B**). We did not find any correlation between the expression of purinergic receptors and the tumor phenotype, probably because of the limited size of our sample.

### Expression Levels of P2Y_2_ Receptor and P2X4 Receptor Are Related to Gastric Cancer Patient Survival

**Figure 8:**
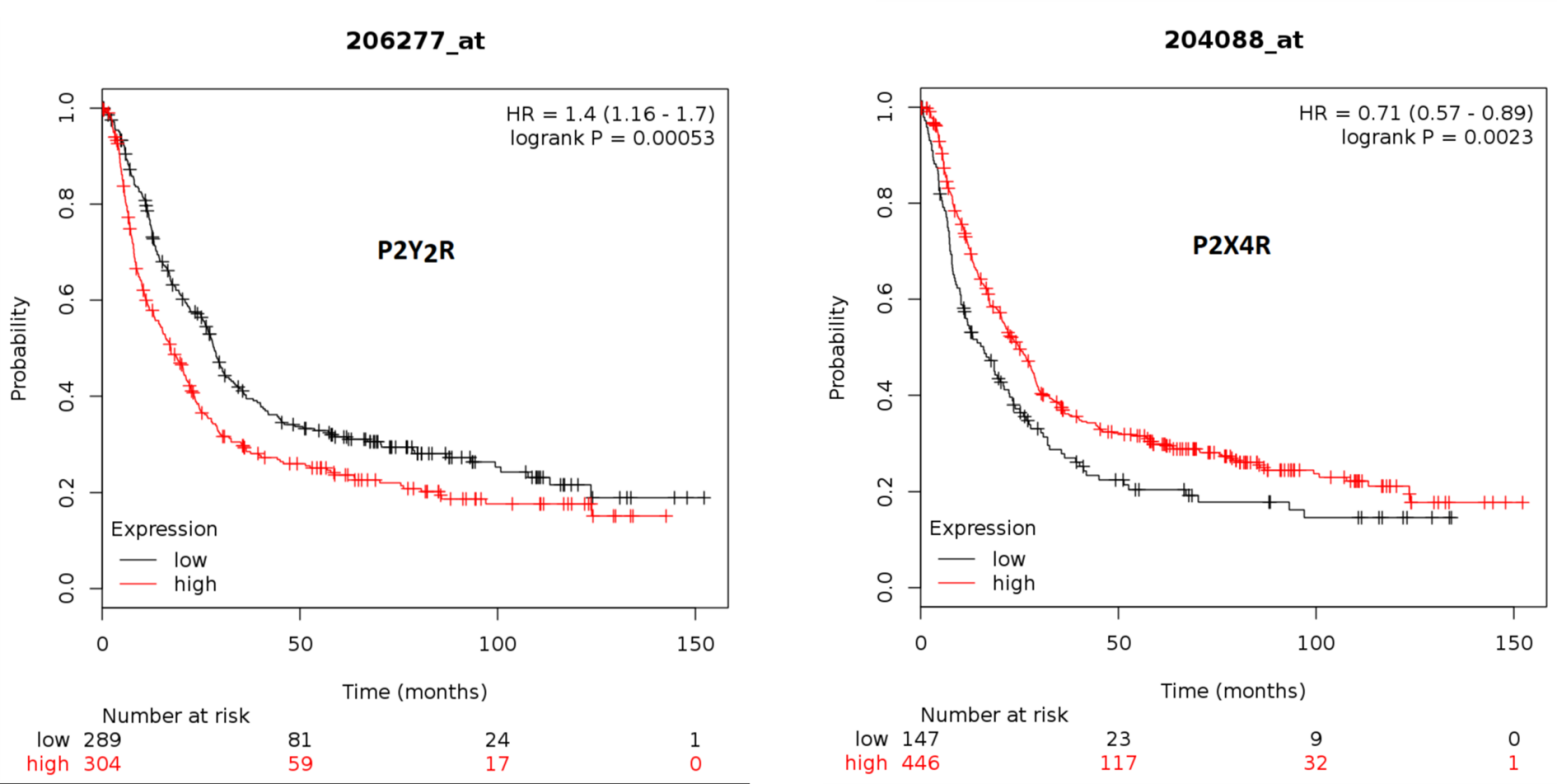
Kaplan–Meier survival plots for high or low expression of P2Y_2_R and P2X4R in GC patients. Database was taken from the Kaplan–Meier Plotter Database (kmplot.com), from a total of 593 patients diagnosed with GC. Plots show the survival of patients with low (black line) and high (red line) expression of the P2Y_2_R (left plot) or the P2X4R (right plot)—number indicates surviving patients at 0, 50, 100, and 150 months.

Finally, we analyzed if high or low expression of P2Y_2_R and P2X4R is related to survival for 593 patients, taken from the Kaplan–Meier Plotter Database (kmplot.com) (Lanczky et al., 2016). Interestingly, we found that patients with high P2Y_2_R expression have a significantly lower survival rate as compared to patients with low P2Y_2_R expression (**Figure 8**). On the other hand, patients with high P2X4R expression have significantly higher survival rates as compared to patients with low P2X4R expression (**Figure 8**).

## Discussion

Gastric cancer (GC) is one of the leading causes of cancer-induced deaths in the world. Although purinergic signaling has been studied in different cancer types, there is not abundant evidence about its role in GC. In the present work we have identified purinergic receptors present in several GC-derived cell lines and also in biopsies from patients diagnosed with GC, finding that the expression pattern varies between the different cell lines, and between healthy and tumor cells, and biopsies. In a first series of experiments, we evaluated the expression of purinergic receptors in three different GC-derived cell lines and in a cell line derived from healthy gastric mucosa. Although the expression varies among the cell lines, we find that healthy mucosa-derived GES-1 expresses more P2XRs as compared to GC-derived cells. In particular, we found that P2X7R and P2X4R are downregulated in GC-derived cell lines; on the other hand, in some GC-derived cell lines, namely, AGS and MKN-74, there is a consistent increase in the expression of P2YRs, especially P2Y_2_R. Consistent with this, we observed in MTT proliferation studies that the activation of P2Y_2_R increases cell viability and proliferation, whereas P2X4R activation decreases it. In our experiments we consistently observed that the P2XR subtype responsible for decreases in GC-derived cell proliferation is P2X4R and not P2X7R, because firstly, we found that P2X7R is almost completely downregulated in AGS and MKN cell lines, and secondly, in cell proliferation studies the P2X7R antagonist AZ10602120 had no effect in preventing the activation of P2XRs by high concentrations of ATP, although we observed an effect of the P2X4R antagonist BX430. Moreover, the P2X4R positive allosteric modulator ivermectin was able to decrease the basal proliferation in AGS cells, and this effect was enhanced in P2X4R-overexpressing AGS cells. We cannot discard the contribution of P2X2R in the ATP-mediated decrease in GC-derived cell proliferation, seeing as we detected the expression of this receptor in AGS and GES-1 cells, but currently there are no reliable antagonists commercially available to test its specific contribution. In addition, we found that the sole application of P2Y_2_R and P2X4R antagonists can change the basal proliferation of these cells, indicating that nucleotides acting as paracrine and/or autocrine signaling molecules can produce an effect. This is especially relevant taking into account that the tumor microenvironment has increased ATP levels (Di Virgilio and Adinolfi, 2017), and this could contribute to tumor progression. We additionally performed calcium imaging experiments to directly asses the activation of P2 receptors and found P2-mediated responses in both AGS and GES-1 cells. In both cell lines the major contribution of intracellular calcium increases was consequence of the activation of P2Y_2_R, although there is also a contribution of P2XRs in GES-1 cells. Three observations support this conclusion: 1) ATP and UTP evoked similar responses that match with the P2Y_2_R pharmacological profile; 2) in AGS cells the ATP or UTP increases in intracellular calcium are comparable in the absence or presence of extracellular calcium suggesting a major contribution of metabotropic receptors; and 3) the responses are sensitive to P2Y_2_R agonist MRS2678 and blocked by P2Y_2_R antagonist ARC-118925XX. All these results highlight the role of purinergic signaling in GC-derived cells and suggest that its pharmacological manipulation could constitute an alternative treatment strategy.

Purinergic signaling plays a role in different types of cancer. For example, studies in a melanoma-derived cell line have shown the presence of P2Y and P2X receptors; in this particular cell line the stimulation of P2Y_1_R decreases the number of cells, whereas stimulation of the P2Y_2_R increases it. Conversely, P2X7R is also present in these cells and its specific activation induces apoptosis (White et al., 2005a; White et al., 2005b). Regarding lung cancer, the derived cell line A549 expresses functional P2Y_2_Rs and P2Y_6_Rs, which mediate cell proliferation after specific activation by purinergic agonists (Schafer et al., 2003). Brain tumors such as gliomas and astrocytomas express P2Y_1_Rs and P2Y_1_2Rs, which in general mediate increases in cell number, but in some cell lines can also induce apoptosis (Sellers et al., 2001; Morrone et al., 2003). In the case of cervical cancer, studies with primary cultures from tumors and cell lines have demonstrated the functional expression of the P2X7R that mediates apoptosis with an expression increase of caspase-3 and caspase-9 (Wang et al., 2004). Proliferation of the breast cancer-derived cell line MCF-7 is decreased with high doses of ATP (over 100 µM), but lower doses of specific activation of P2Y_2_R increase cell proliferation, probably reflecting the action of different P2 receptor subtypes (Slater et al., 2004). More recently, it has been suggested that activation of P2Y_2_Rs also increases MCF-7 cell migration through the MEK-ERK1/2 signaling pathway (Chadet et al., 2014). In ovarian cancer, there are also conflicting studies: one group has reported an increase in cell proliferation on the OVCAR-3 cell line mediated by P2Y_2_R (Popper and Batra, 1993), whereas another group has found opposite effects mediated by this receptor in the EFO-21 and EFO-27 cell lines (Schultze-Mosgau et al., 2000). More recent works have addressed a role of purinergic signaling and P2Y_2_R in the regulation of cell migration and epithelium to mesenchymal transition in ovarian carcinoma-derived cells (Martinez-Ramirez et al., 2016). In leukemia, the roles of functional P2X7Rs and P2Y_11_Rs in lymphocytes from patients suffering this disease have been demonstrated, and it has been proposed that P2X7R mediates cell death, while P2Y_11_R mediates cell differentiation (Conigrave et al., 2000; Zhang et al., 2004). As in other cancers, conclusions about the role of purinergic signaling in colorectal cancer are not uniform. In one study, cell proliferation was increased by the activation of P2Y_2_R in primary cultures from colorectal tumors and in the HT29 cell line (Hopfner et al., 1998). However, another study showed an increase of proliferation mediated by the P2Y_2_R and a decrease in cell number mediated by P2Y_1_Rs and P2X7Rs in HCT8 and Caco-2 cell lines (Coutinho-Silva et al., 2005). In cells derived from human esophageal cancer, it has been shown that the expression of P2Y_2_Rs and P2X4Rs and the activation of the former leads to a decrease in cell number (Maaser et al., 2002). In human cancerous pancreatic duct epithelial cells, it has been also reported that the activation of P2Y_2_R by UTP increases cell proliferation and this process is mediated by the PI3K/Akt pathway (Choi et al., 2013). In prostate cancer, it has been shown that P2Y_2_R cooperates with EGFR to promote cell invasion and metastasis through the ERK1/2 pathway, suggesting a therapeutic possibility using purinergic blockers to treat this type of cancer (Li et al., 2015). Recently it has also been reported that platelet-derived nucleotides that activate an endothelial P2Y_2_R, is a mechanism that allows metastasis of tumor cells by transendothelial migration (Schumacher et al., 2013). However, in GC there is not much information available regarding purinergic signaling. The first studies in this field have reported that adenosine and ATP could inhibit cell growth and induce apoptosis in GC-derived cell lines GT3-TKB and HGC-27, acting mainly through P1 receptors (Saitoh et al., 2004; Wang and Ren, 2006).

As in other types of cancer, in GC a change in the expression of several genes has been observed. One example is the overexpression of the gene encoding the human epidermal growth factor receptor 2 (HER2) found not only in GC, but also in other cancers such as colorectal, lung, and ovarian cancer (Gravalos and Jimeno, 2008). Other genes that are overexpressed in GC are ones that codify for the epidermal growth factor receptor (EGFR), vascular endothelial growth factor A (VEGFA) and its respective receptor, fibroblast growth factor receptor (FGFR), and hepatocyte growth factor receptor (Carcas, 2014). In addition, a variety of molecules that participate in events such as cell cycle or control of cell adhesion have been proposed as early biomarkers for GC and can be detected from tissues of fluids using molecular biology techniques (Yasui et al., 2011). These biomarkers include ADAM17 (Shou et al., 2012), MMP2, MMP9, and MMP11 (Sampieri et al., 2010; Zhao et al., 2010), survivin and CK19 (Bertazza et al., 2009), vimentin (Iwatsuki et al., 2010), CXCR4 (Ingold et al., 2010), ING5 (Xing et al., 2011), and Stanniocalcin2 (Yokobori et al., 2010); in all of these examples, these molecules are overexpressed in tumoral GC tissues. However, these changes are not uniform, and therapy effectiveness will depend on the degree of overexpression of the target gene. Although several pharmacological therapies for the treatment of GC have been studied, only two targeted treatments have been approved in the United States after positive clinical trials. One of these treatments is an HER2 inhibitor and has been studied as a therapy for several cancers. This drug, which has been termed trastuzumab, is a monoclonal antibody that binds to HER2 inhibiting signaling and preventing the cleavage of its extracellular domain (Hudis, 2007). However, trastuzumab treatment is only effective in patients with scores of IHC 2+ (equivocal) or IHC 3+ (positive) of HER2 overexpression (Hofmann et al., 2008). For that reason it is relevant to find new therapeutic targets, especially for patients with low HER2 scores.

In our GC experiments, we found that purinergic signaling exerts different effects that depend in the expression profile of purinergic receptors of the cell lines; for example UTP induces proliferation of AGS and MKN-74 cells, and both cell lines show a strong expression of the P2Y_2_R, while the same agonist has no effect on MKN-45 cells that show a low expression of this receptor. This reflects the high variability among cancer and highlights the importance of a detailed disease characterization for each patient. Concordantly, in a last series of experiments we evaluated purinergic receptors in human GC biopsies and compared their expression of the tumor and their nearby healthy gastric mucosa. Although in general we found that the expression of P2Y_2_R is increased and P2X4R is decreased in GC, when we compared the results of seven different patients, we found that these changes are not uniform. We infer that similarly to what has been observed with HER2 overexpression, patients with a high purinergic component will be more sensitive to a purinergic treatment. Interestingly, when we analyzed the survival of GC patients in terms of the expression of P2Y_2_R and P2X4R, we observed a positive correlation between their high or low expression and the survival rates; whereas high expression of P2Y_2_R decreased the survival of GC patients, high expression of P2X4R increased their survival. In this context, the grade of expression of some purinergic receptors, such as P2Y_2_R or P2X4R, could constitute a potential biomarker for GC.

In summary, we have described for the first time the purinergic signaling in GC-derived cell lines and found that purinergic signaling is a complex event that depends on the expression profile of specific purinergic receptors and on the nucleotide concentration present in the extracellular environment. The inhibition of some key purinergic receptors, for example P2Y_2_R, plus the activation or other subtypes, for example P2X4R, could constitute a future strategy for GC treatment.

## Data Availability Statement

All datasets generated for this study are included in the manuscript and the supplementary files.

## Ethics Statement

The protocols were approved by the Ethical-Scientific Committee of the Faculty of Medicine of the Universidad Católica del Norte (CECFAMED-UCN) and patients voluntarily signed their consent, document F.M. Nº 17.

## Author Contributions

CC, PC, MH, EF-O, and GB designed research; MH, PC, FR-T, CR-P, SR-R, FG, and EF-O performed experiments; MJH, PC, and CC analyzed data; JAM, FG, JL, and MB provided human biopsies; and CC, PC, and MH wrote the manuscript. KP-I and MR-J performed experiments and analyzed data.

## Funding

This work was funded by Fondo Nacional de Desarrollo Científico y Tecnológico (FONDECYT) Regular Grant # 1161490, CONICYT Ph.D. Fellowship #21181885 and FONDEQUIP EQM140100. The funders had no role in the study design, data collection and analysis, decision to publish, or preparation of the article.

## Conflict of Interest Statement

The authors declare that the research was conducted in the absence of any commercial or financial relationships that could be construed as a potential conflict of interest.
