# Supplementary material for: Differential Effects of Purinergic Signaling in Gastric Cancer-Derived Cells Through P2Y and P2X Receptors
Source: Front Pharmacol. 2019 Jun 13;10:612. doi: 10.3389/fphar.2019.00612 (PMC6584115; doi:10.3389/fphar.2019.00612)
Supplement: Supplementary file 1 [file DataSheet_1.pdf]

Supplemental figure 1

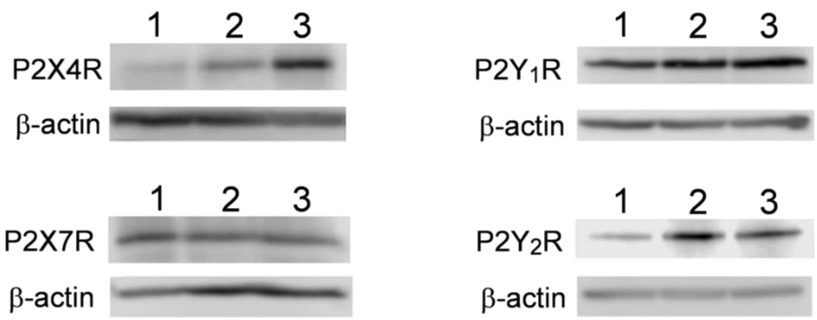

Representative blots of the expression of different P2 receptors in GES-1 cells cultures after 24(1), 48(2) or 84(3) hours of plating.

Supplemental figure 2

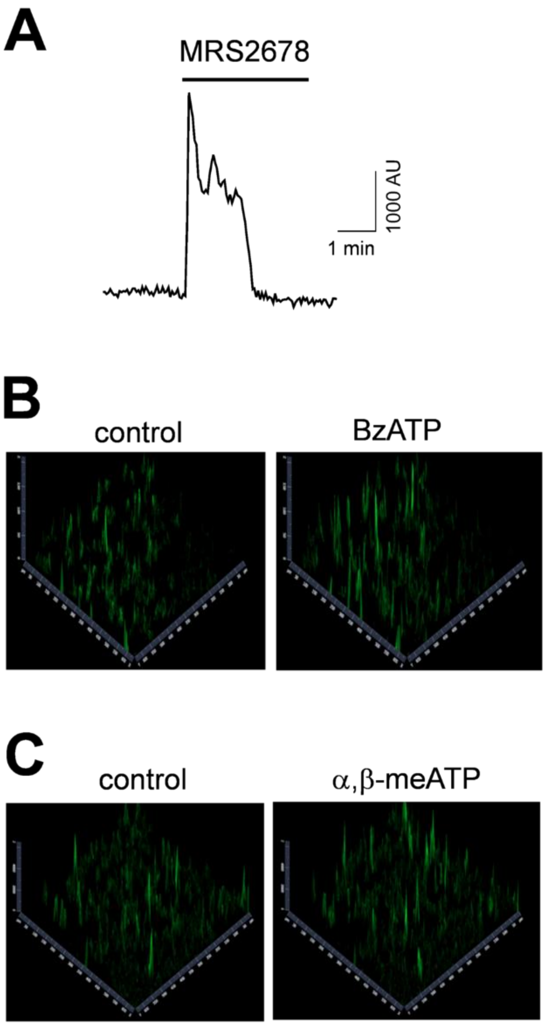

A. Representative recording from single AGS cell showing the increase of [Ca2+]i induced by 10 μM MRS2678, a P2Y2R-specific agonist. **B and C.** Representative 2.5D of the effects of application of 100 μM BzATP (**B**) or α,β-meATP (**C**) to AGS cells.

Supplemental figure 3

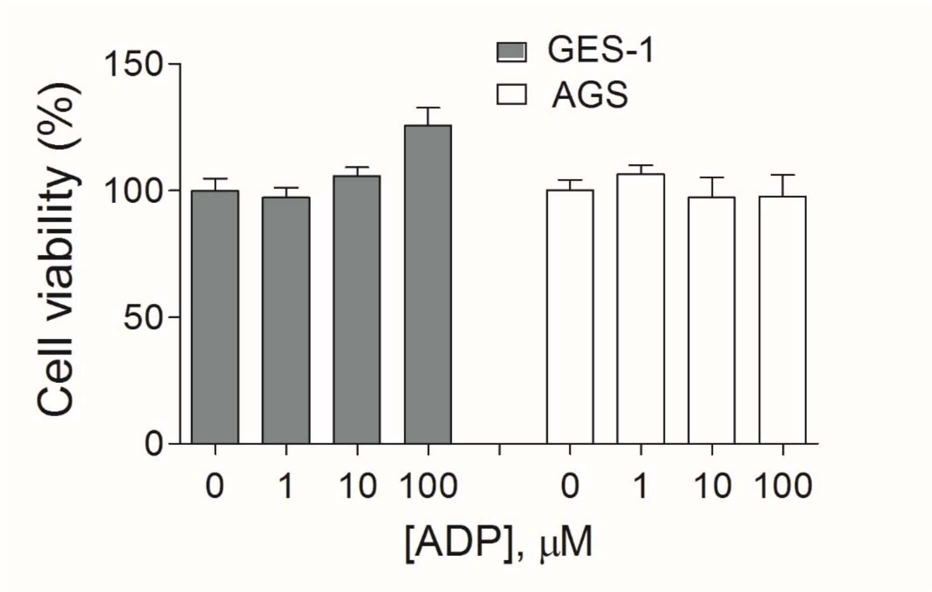

Summary of cell viability experiments on AGS cells showing the effect of 1-100 μM ADP on GES-1 (grey bars) or AGS (open bars) cells, n = 3.
